# Gluc-HET, a complementary chick embryo model for the characterization of antidiabetic compounds

**DOI:** 10.1371/journal.pone.0182788

**Published:** 2017-08-04

**Authors:** Renate Haselgrübler, Flora Stübl, Katja Essl, Marcus Iken, Klaus Schröder, Julian Weghuber

**Affiliations:** 1 University of Applied Sciences Upper Austria, Wels, Austria; 2 PM International AG, Schengen, Luxembourg; 3 NP Life Science Technologies KG, Linz, Austria; 4 Austrian Competence Center for Feed and Food Quality, Safety and Innovation, Wels, Austria; Tohoku University, JAPAN

## Abstract

Insulin resistance and β cell failure are the main causes of elevated blood glucose levels in Type 2 diabetes mellitus (T2DM), a complex and multifactorial metabolic disease. Several medications to treat or reduce the symptoms of T2DM are used, including the injection of insulin and the application of insulin sensitizing or glucose production reducing drugs. Furthermore, the use of phytochemicals has attracted increasing attention for the therapy and prevention of T2DM. In order to identify and characterize antidiabetic compounds, efficient test systems are required. Here we present a modified chick embryo model (hens egg test, HET), which has originally been developed to determine the potential irritancy of chemicals, as a versatile tool for the characterization of phytochemicals with antidiabetic properties. We termed this modified assay variation *Gluc-HET*. More precisely, we determined the influence of variations in the incubation time of the fertilized eggs and studied the effects of different buffer parameters, such as the temperature, composition and volume, used for drug application. In addition, we tested several putative antidiabetic plant extracts, which have been identified in an *in-vitro* primary screening procedure, for their effectiveness in reducing blood glucose levels *in-ovo*. Taken together, our Gluc-HET model has proven to be a reliable and manageable system for the characterization of antidiabetic compounds.

## Introduction

Diabetes mellitus is a group of metabolic diseases characterized by hyperglycemia resulting from defects in insulin secretion, insulin action, or both [1]. In type 2 diabetes mellitus (T2DM), which accounts for ~90–95% of the diabetes cases, individuals have insulin resistance and usually a relative insulin deficiency [2]. Changes in lifestyle in the last decades, especially those related to overnutrition, physical inactivity, and aging are known factors increasing the global incidence of T2DM. According to the International Diabetes Federation (IDF), nearly 400 million people have diabetes mellitus worldwide, and this number is expected to reach 600 million by 2035 [3]. Insulin resistance is the basis for a number of chronic diseases such as hypertension, dyslipidemia, glucose intolerance, coronary heart disease, and cerebral vascular disease along with T2DM [4]. Its prevalence has been increasing steadily all over the world, and T2DM is becoming a serious problem regarding the financial burden for health care systems. For example, the total estimated economic cost of diagnosed diabetes in the U.S. only is $245 billion [5].

Diabetic complications caused by hyperglycemia decrease the life quality and expectancy significantly. In addition to lifestyle modifications, most diabetic individuals eventually require glucose-lowering pharmacotherapy such as metformin [6] or glitazone [7] therapy. However, application of these drugs is frequently associated with side effects including diarrhea, nausea, abdominal pain, edema and congestive heart failure, which limits their use in T2DM patients [8–10]. Therefore, additional safe, widely available and inexpensive anti-diabetic approaches are required. Several plants that are consumed in traditional Chinese medicine or as nutraceuticals contain phytochemicals that have antidiabetic effects through the modulation of diverse cellular and physiological pathways, including modulation of lipid absorption and metabolism, enhancement of insulin sensitivity, inhibition of intestinal glucose uptake, and stimulation of glucose transporter 4 (GLUT4) translocation leading to increased glucose uptake in muscle and adipose tissue [11].

The induction of GLUT4 translocation in the absence of insulin by phytochemicals, known as insulin mimetic compounds, is a reasonable strategy to combat the effects of insulin resistance. In theory these compounds should lower blood glucose levels by increasing the number of GLUT4 proteins in the plasma membrane of adipocytes and muscle cells and also in the absence of insulin or a reduced insulin sensitivity of these cells. We have developed a highly sensitive fluorescence microscopy-based approach to quantify the translocation process of GLUT4 [12] and are currently using this assay to test herbal extracts for their GLUT4 translocation-inducing properties. A plant extract collection containing more than 4,500 unique extracts [13] is currently being screened for putative insulin mimetic phytochemicals. Primary hits are verified by wet lab chemistry approaches, but tests in living organisms are of pivotal importance.

For this purpose, we decided to modify the hens egg test (HET) on the basis of the well-established hens egg test-chorioallantoic membrane (HET-CAM) assay. It is an alternative test system to animal testing widely used in industry, and it can be used instead of the rabbit eye irritation test to determine and evaluate irritating properties of compounds and formulations [14]. Furthermore, the HET is widely used in pharmacological and basic research as an alternative assay to animal experimentation. For example the high vascularization of the CAM enables the analysis of angiogenesis [15], the genotoxic properties of test compounds [16] or the teratogenic potential of crop protectants [17].

Experiments performed with non-hatched avian embryos in the first two-thirds of embryonic development time are not considered an animal experiment according to the Directive 2010/63/EU. HET is an alternative to tests with live animals such as the Draize rabbit eye test. An approval by an ethics committee is not required since the HET-CAM test is not an animal experiment in a legal sense. The avian egg is a closed system, where the embryo develops only by the exchange of heat, respiratory gases and water vapor with its environment. The complete development requires 21 days until the embryo is ready for hatching. All nutrients including glucose, amino acids, vitamins, trace elements, lipids, and proteins are stored in the egg before it has been laid [18]. The development of the pancreas starts at day 5 of incubation and then develops until day 12, when it is finally in a functional state. Insulin production starts at day 5 but increases until day 12. Serum insulin levels could not be measured until day 12 of development [19]. Accordingly, interference between naturally produced insulin and insulin mimetic compounds applied until day 12 can be regarded as non-relevant. Therefore, the chick embryo seems to be a very promising model, and we have already successfully used it for the characterization of selected herbal extracts [20].

Here we describe the modified HET-CAM assay to study insulin mimetic compounds in detail, which we call Gluc-HET. We present data describing the temporal resolution of the test system, describe the effects of variations in the duration of embryo incubation (10 and 11 days, respectively), and focus on the variation of buffer parameters that are used for dissolving the extracts. NovoRapid, a rapid acting insulin analog, was used as a positive standard. Finally, we present data on several herbal extracts, which were tested with the Gluc-HET assay. The results presented confirm the versatility and eligibility of the approach to test the putative blood glucose lowering effects of compounds in a living organism.

## Materials and methods

### Chemicals

Phosphate buffered saline (PBS), Hank’s balanced salt solution (HBSS), N-2-hydroxyethylpiperazine-N-2-ethane sulfonic acid (HEPES) and Krebs-Ringer-phosphate-HEPES (KRPH) were purchased from Sigma-Aldrich (Schnelldorf, Germany). NovoRapid manufactured by Novo Nordisk was a kind gift from Daniel Weghuber (Paracelsus Medical University, Salzburg, Austria).

### Hens egg test for blood glucose levels

Fertilized hens eggs (Lohmann classic brown chicken) were obtained from a local breeder. Storage of the eggs was done at 14°C in a humidified area. The eggs were stored no longer than 10 days after laying and were finally incubated at 38°C with an average humidity of 40–60% for 10 or 11 days, respectively. Based on our observations, day 11 corresponds to stage 35–36 in the development of the chick embryo, as described by Hamburger and Hamilton [21]. The eggs were automatically and constantly turned, checked for fertilization via candling, and the area of the air bladder was marked. The eggshell was lightly pecked with a pointed pair of tweezers in this area and 100 or 300 μL of a buffer solution (HBSS, PBS or HEPES) containing the putative blood glucose-lowering substance was added. We tested different water soluble plant extracts obtained from an extract library. The stock concentration of the extracts was defined to be approximately 10 g/L [13]. HBSS buffer was used to dilute the extracts to a final concentration of 300 mg/L, which was finally applied with a syringe into the air compartment of the egg. The eggs were placed back in the incubator for different time intervals (up to 3 h) to allow the test substances to soak through the eggshell membrane and get in close contact with the chorioallantoic membrane. After incubation for different time intervals, the eggshell above the air bladder was carefully removed, and the eggshell membrane was equilibrated with PBS. In the next step, the eggshell membrane was removed, and the chorioallantoic membrane was carefully cut with a micro-scissor. This is done to gain better access to a suitable blood vessel for blood collection. At this step, it is very important to avoid the cutting of large vessels in the membrane itself, which would lead to a non-preferable loss of blood. A suitable blood vessel was carefully placed on a plastic pH strip, which is patted dry using filter paper before the vessel is cut, and leaking blood (10–500 μL, depending on blood glucose quantitation method) is collected. The blood glucose levels were determined via a blood glucose meter (Accu-Check Performa, Roche Diabetes Care GmbH, Mannheim, Germany) or HPLC as previously reported [20]. For each time point, at least 10 fertilized eggs were used. Each experiment was repeated at least two times. The rapid loss of blood during blood collection led to an immediate death of the chicken embryo. No additional manipulations to the fertilized eggs occurred after the 11^th^ day of incubation time.

### Data analysis

Statistical analysis was carried out using the unpaired t-test in Graphpad Prism (version 6.02). Figures were prepared using Corel Draw (version X6).

## Results

### Description of the Gluc-HET system

**Fig 1:**
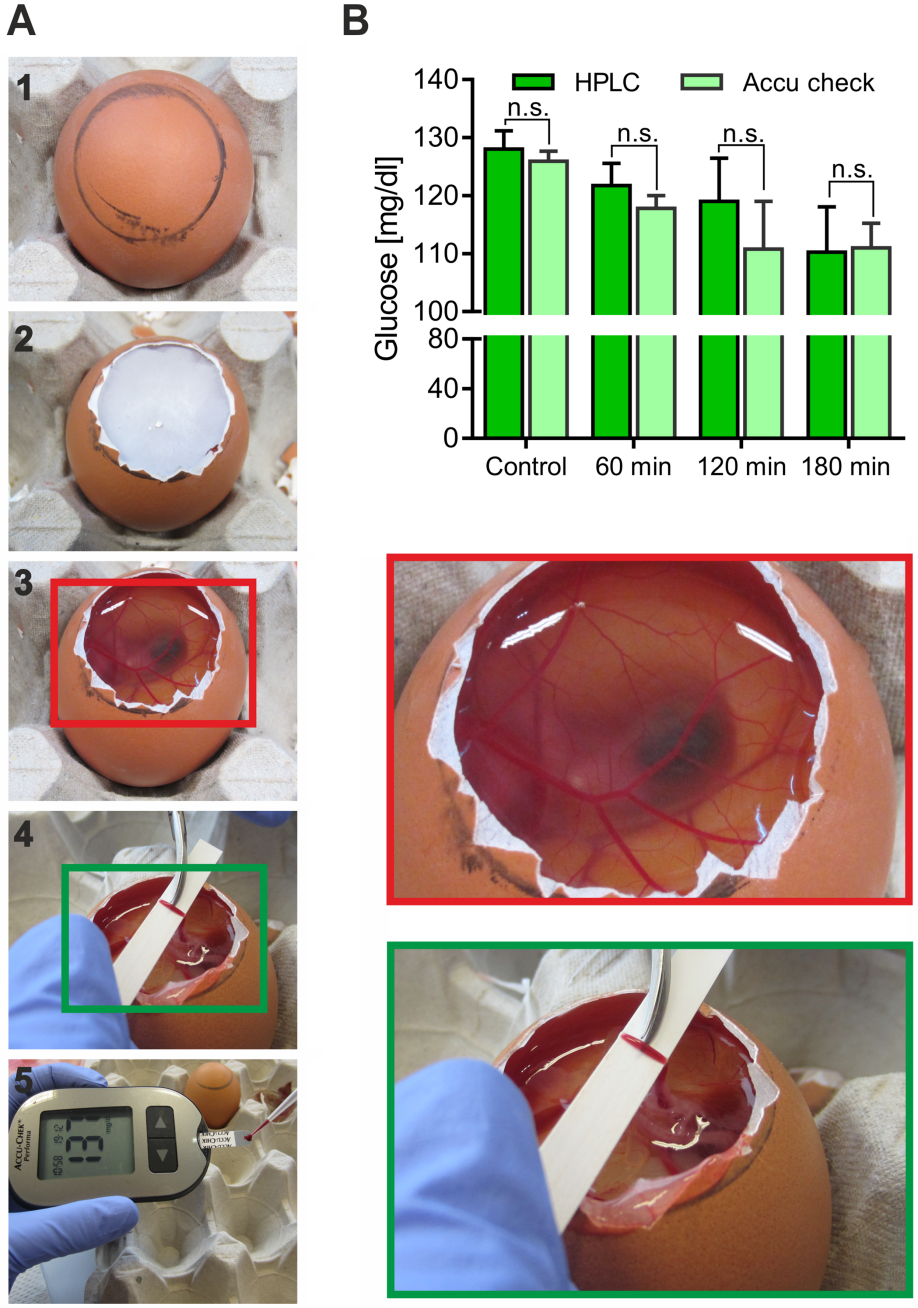
Description of the Gluc-HET model. (A) Important steps of a Gluc-HET experiment. From top to bottom: opening and removal of the eggshell (1–2); equilibration and removal of the eggshell membrane with the chorioallantoic membrane exposed (3); prepared vessel on a pH strip (4); and collection of blood and measurement of the glucose concentration by a glucose meter (5). The enlarged images (red and green rectangles) highlight the nature and handling of the blood vessels. (B) Comparison of two methods to quantitate blood glucose levels. Eggs were incubated without (control) or with KRPH buffer for different incubation times, and the blood glucose was determined by HPLC or with a blood glucose meter. Error bars are based on the standard error of the mean.

After incubation, the fertilized eggs are carefully opened and the eggshell is removed (Fig 1A). Upon equilibration with PBS buffer, the eggshell membrane is removed to gain access to the chorioallantoic membrane, which is then cut. In the next step, a suitable blood vessel is carefully placed on a plastic pH strip, which is patted dry using filter paper before the vessel is cut, and leaking blood (300–500 μL) is collected. The patting process is a crucial step to avoid dilution effects of the present equilibration buffer solution, which would falsify the estimated blood glucose concentration. For quantitation of the blood glucose levels, we tested two different methods, HPLC and a blood glucose meter, respectively. HPLC proved to be highly sensitive; however, the experimental procedure is very laborious. Therefore, we tested a blood glucose meter in combination with suitable test strips. As shown in Fig 1B, both experimental procedures resulted in comparable glucose concentrations with no significant differences. Taken together, a fast and less expensive quantitation using a blood glucose meter is reasonable.

### Influence of the embryo developmental stage on assay performance

**Fig 2:**
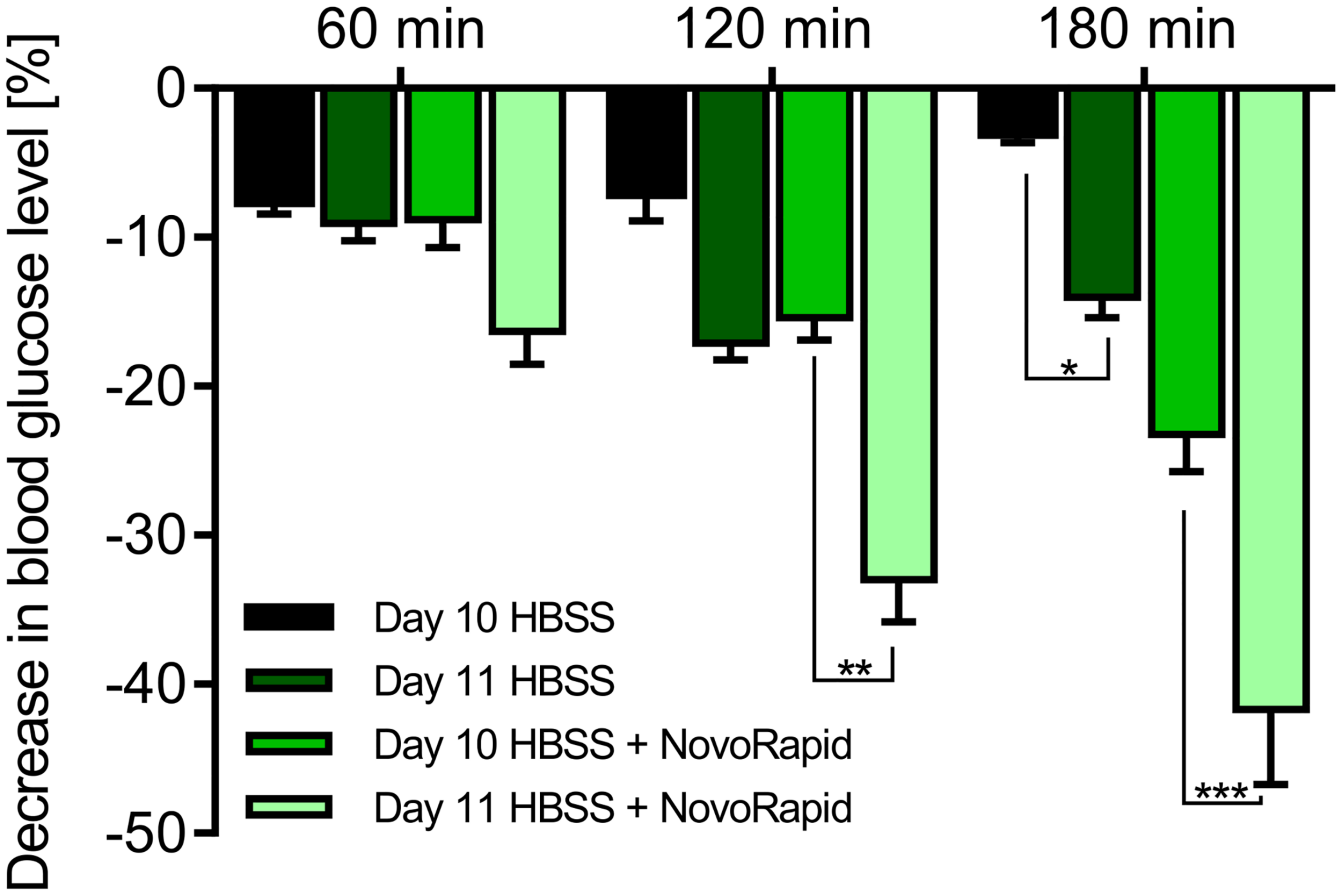
Influence of the embryonal development stage (10 versus 11 days) on Gluc-HET performance. Comparison of experiments performed with eggs incubated for 10 and 11 days, respectively. Eggs were treated with HBSS buffer with or without NovoRapid (3.3 U/mL) for up to 3 hours. Error bars are based on the standard error of the mean. *P < 0.05, **P< 0.01 and ***P < 0.001, significant difference between 10 and 11 days.

For most of our previous experiments, we incubated the eggs for 10 days. However, the performance on day 10 is not optimal, and many embryos are lost during the preparation process. The handling of the embryos is challenging due the small size and fragility of the blood vessels. We therefore decided to increase the incubation time to 11 days. In general, the embryos and their blood vessels are larger and less fragile then. We tested the functionality of the Gluc-HET assay by comparing the blood glucose lowering effects of NovoRapid using embryos aged 10 and 11 days, respectively. NovoRapid, which is a human rapid acting insulin analog, was used as a positive control in all experiments for the reduction of blood glucose levels. First, we determined the influence of the buffer usually used for compound dilution. As shown in Fig 2, HBSS buffer led to a minor decrease of -7.1% after 2 hours of incubation time in embryos aged 10 days. Under the same experimental conditions, embryos treated with NovoRapid (3.3 U/mL) exhibited a decrease of -8.8%, -15.4% and -23.2% after 1, 2 and 3 hours of incubation time, respectively. In embryos aged 11 days, HBSS buffer resulted in a more pronounced decrease of blood glucose levels of -17.11% after 2 hours of incubation time. However, under the same experimental conditions, NovoRapid-treated embryos exhibited decreases of -16.3%, -33% and -41.7% after 1, 2 and 3 hours of incubation time, respectively. In conclusion, embryos aged 11 days are more sensitive to buffer exposure. However, the efficacies of known blood glucose-reducing compounds such as NovoRapid (Fig 2) are also further increased. We therefore consider an extended embryo incubation time of 11 days as being preferable.

### Influence of buffer parameters on assay performance

**Fig 3:**
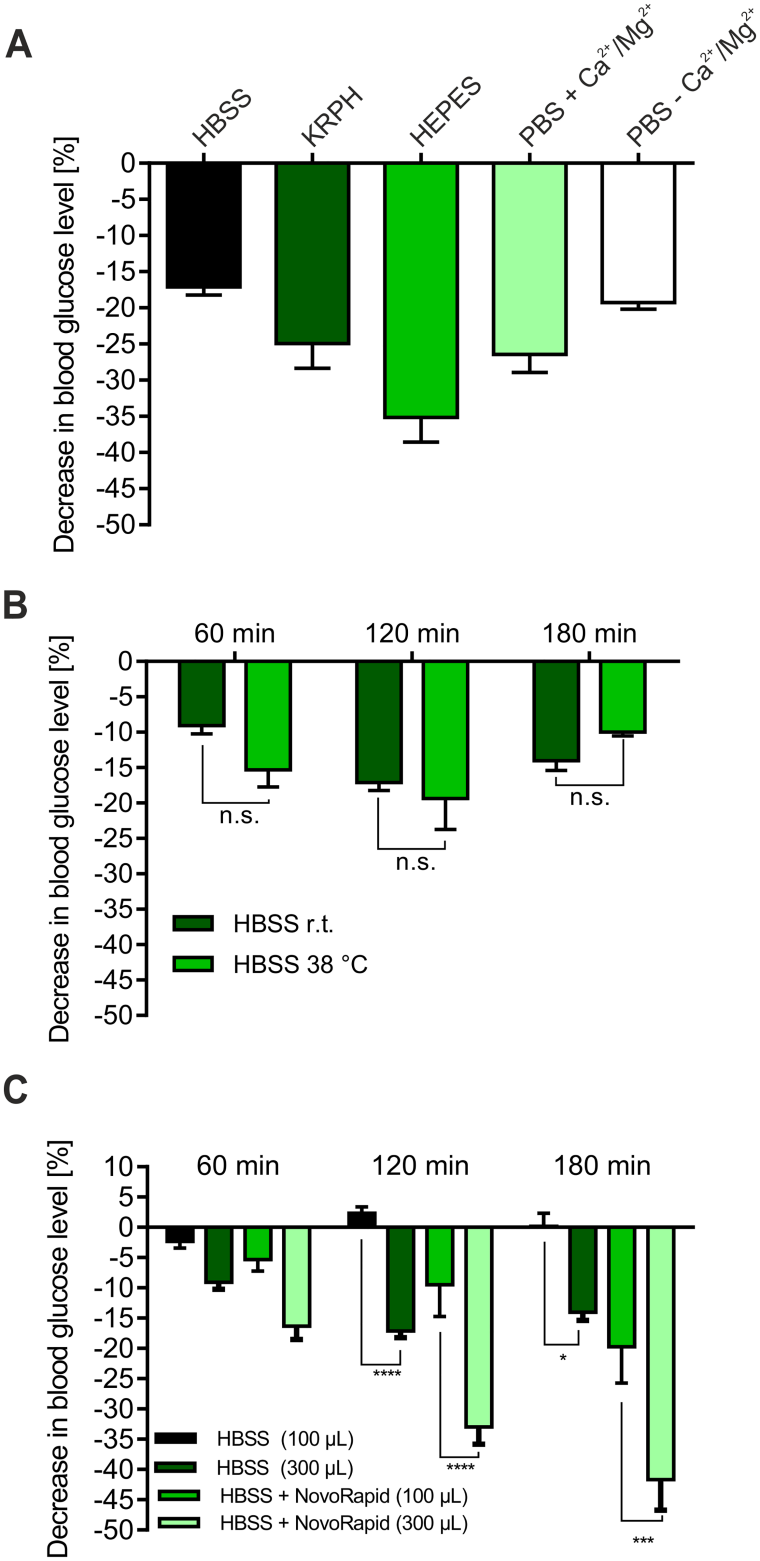
Influence of the buffer parameters on the Gluc-HET performance. (A) Comparison of different buffer systems. Eggs were incubated for 11 days and treated with different buffer formulations for 120 min. The drop of the blood glucose level was determined via a blood glucose meter. Error bars are based on the standard error of the mean. (B) Influence of the buffer temperature on the assay performance. Eggs were incubated for 11 days and treated either with HBSS buffer stored at 38°C or room temperature (23°C). Blood glucose levels were determined by a blood glucose meter. Error bars are based on the standard error of the mean. (C) Influence of the buffer volume on the assay performance. Eggs were incubated for 11 days and treated with either 100 μL or 300 μL of HBSS with or without NovoRapid (3.3 U/mL) for up to 3 hours. Blood glucose levels were determined by a blood glucose meter. Error bars are based on the standard error of the mean. *P < 0.05, ***P < 0.001 and ****P < 0.0001, significant difference between 100 μL and 300 μL.

Due to the observed blood glucose-reducing effect of buffer exposure alone, especially in embryos aged 11 days, we decided to test different buffer formulations. Thus, we determined the influence of KRPH, PBS with/without Mg^2+^ and Ca^2+^, HBSS and HEPES buffers in embryos aged 11 days. As shown in Fig 3A, HBSS buffer resulted in the lowest decreases of -9.1%, -17.1% and -14% after 1, 2 and 3 hours, respectively. PBS buffer, independent of the presence of Mg^2+^ and Ca^2+^, KRPH and especially HEPES buffer led to significant decreases between -25% and -35% after 2 hours of incubation time. Thus, the usage of HBSS buffer for the application of the antidiabetic compounds appears straightforward.

To test whether the buffer temperature influences the results obtained by the assay, eggs were incubated with HBSS buffer stored at room temperature (23°C) or at 38°C prior to experiments. As shown in Fig 3B, the buffer temperature did not influence the results.

Finally, we quantified the putative effect of the buffer volume used on the assay performance. Therefore, we treated eggs incubated for 11 days either with 100 μL or 300 μL HBSS including or lacking NovoRapid (3.3 U/mL). As shown in Fig 3C, this parameter was confirmed to be an important one, significantly influencing the determined blood glucose levels. A reduction of the used buffer volume to 100 μL resulted in a much lower decrease compared to 300 μL (-2.3%, 2.3% and 0.2% for 100 μL of HBSS vs. -9.1%, -17.1% and -14% for 300 μL of HBSS, respectively). This also holds true for HBSS including NovoRapid, especially after 2 and 3 h of treatment (-5.2%, -10.6% and -20.2% for 100 μL HBSS + NovoRapid, respectively). Based on these results, a reduced buffer volume might be advantageous.

### Effects of selected herbal extracts on blood glucose levels

**Fig 4:**
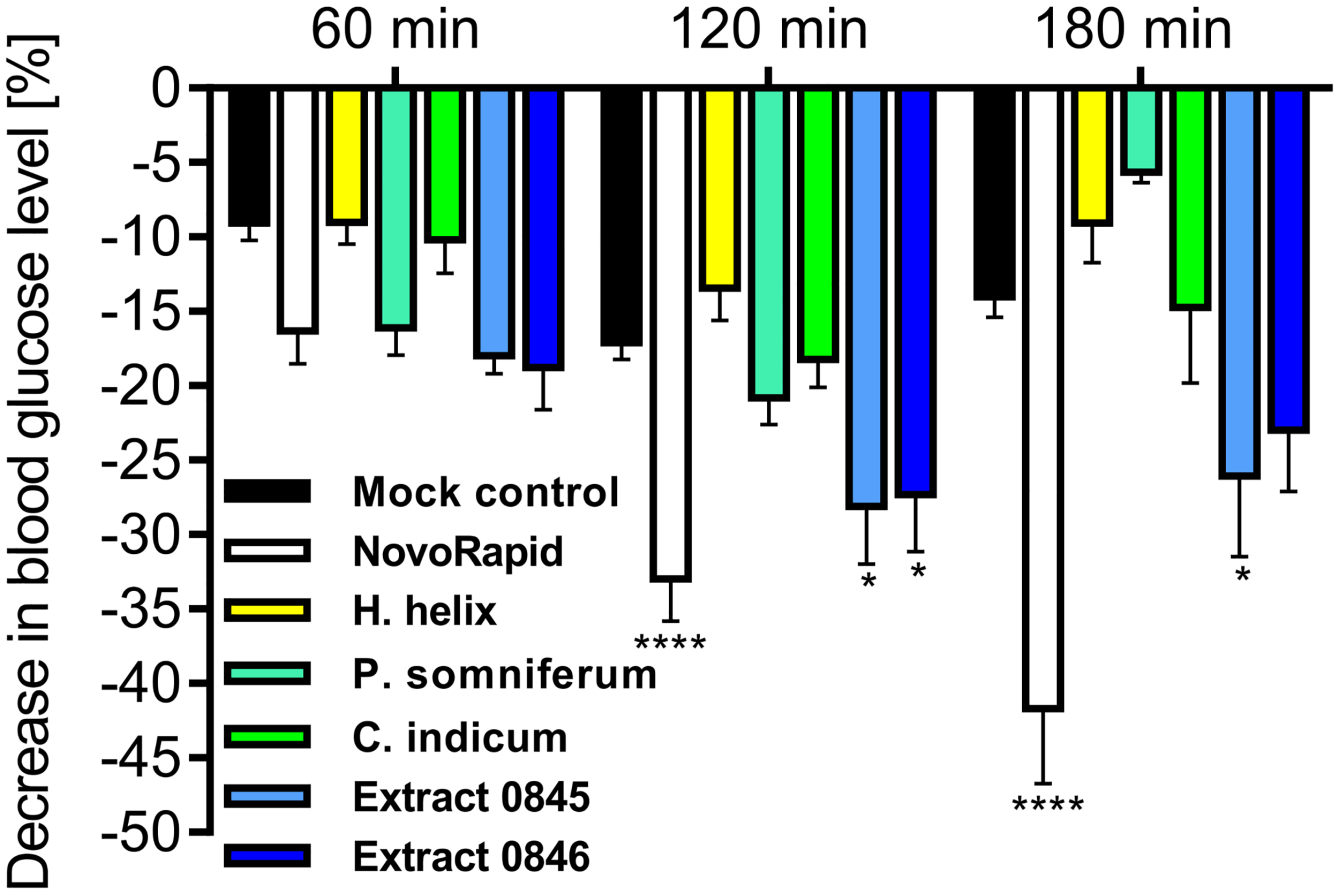
Influence of various herbal extracts on blood glucose levels. Eggs were incubated for 11 days and treated with the indicated substances (NovoRapid: 3.3 U/mL; extracts: 300 mg/L) dissolved in HBSS buffer (300 μL volume) for up to 3 hours. Blood glucose levels were determined with a blood glucose meter. Error bars are based on the standard error of the mean. *P < 0.05 and ****P < 0.0001, significant decrease with respect to HBSS-treated eggs of the same incubation time.

A GLUT4-translocation quantitation based primary screen established in our lab [12] of 2,300 water soluble herbal extracts [13] led to the identification of several hits, which we tested by using the Gluc-HET assay. Extracts prepared from *Hedera helix* (ivy), *Papaver somniferum* (poppy), *Combretum indicum* (Rangoon creeper) and 2 extracts that cannot be disclosed at this time (termed extracts 0845 and 0846) were dissolved in HBSS buffer (300 mg/L) and applied to the embryos aged 11 days. These herbal extracts were used because they were found to significantly induce the translocation and plasma membrane insertion of GLUT4 in CHO-K1 cells [12]. Importantly, all applied extracts did not negatively influence the survival rate of the developing embryos. We incubated the eggs with all tested herbal extracts for 24 hours, opened the eggs and checked the embryos for vitality and potential lesions of the blood vessels in the chorioallantoic membrane. No toxic effects were found in comparison to untreated eggs. Thus, cytotoxic effects that might interfere with the blood glucose concentration can be excluded within this time period. As shown in Fig 4, extracts prepared from ivy, poppy and Rangoon creeper did not result in a significant blood glucose reduction *in-ovo*. While a minor decrease might be observable after 60 min of incubation time for poppy-treated embryos, this effect disappeared after 2 and 3 hours. Interestingly, two related extracts (0845 and 0846, respectively) successfully reduced the blood glucose concentration. These compounds led to a minor decrease after 60 min, comparable to the effect of NovoRapid, and a significant effect after a prolonged incubation time. In summary, the herein described *in-ovo* test is a promising system to verify the efficacy of compounds with identified *in-vitro* activity.

## Discussion

The impact of diabetes on human health and the global health care systems is a dramatic one. The predicted increase of affected individuals is serious and needs to be solved. Current strategies are either expensive and exhausting or characterized by massive side effects. Therefore, new approaches are required to overcome the problem or at least improve this medicinal crisis situation, especially considering the combination with emerging obesity [22].

A promising strategy is the application of insulin mimetic compounds, which induce the translocation of GLUT4 to the plasma membrane [23], also in the absence of insulin, and thereby reduce the blood glucose concentration. Synthetic compounds [24], minerals [25] and herbal substances provided by nature might be useful in this regard [26].

There are several approaches to test the efficacy of such compounds *in-vitro* and in live cells [27, 28], and they also need to be tested with appropriate through-put rates [12]. However, the putative activity of such compounds *in-vitro* needs to also be validated in a living organism. Human clinical studies are extremely time-consuming and are considered only for well-characterized compounds. Studies based on live animals such as mice are important but suffer from low through-put rates and high costs.

The *in-ovo* Gluc-HET system described here represents a promising tool to fill the gap between *in-vitro* and *in-vivo* approaches. It offers several advantages such as acceptable costs and adequate through-put rates (6–8 compounds per week including all controls; 3 experimental days) in addition to its status as a non-animal method. Experiments with non-hatched avian embryos in the first two-thirds of embryonic development time are not considered to be animal experiments according to the Directive 2010/63/EU. This is based on the fact that an intact nervous system is not developed before day 11, which is the maximum age of embryos used for the experiments presented here [18]. We treated the embryos like fetal forms of mammals and terminated our experiments no later than day 11, which does not fall into the last third of their development, which lasts 21 days. Importantly, the chick embryo aged 10 or 11 days is characterized by high glucose levels [18]. In addition, it is susceptible to insulin, although insulin production starts no earlier than day 12 [19]. Accordingly, interference between naturally produced insulin and insulin mimetic compounds applied until day 12 can be regarded as non-relevant.

Previously, we have successfully used the Gluc-HET system to validate the activity of a few selected insulin mimetic herbal compounds [20]. However, the performance and reproducibility of the approach did not fully meet our expectations. Therefore, we performed several experiments to better characterize the Gluc-HET assay. The most important findings described in this study are as follows:

i) The handling of embryos aged 11 days is significantly better than with embryos aged 10 days. The difference in embryo age of a single day might seem minor. However, the blood vessels are more robust and larger, and therefore, the collection of blood (up to 600 μL) is tremendously easier. In addition, the drop-out rate of embryos dying during the incubation process is significantly lower.

The great amount of collected blood enables the quantitation of blood glucose levels by HPLC. In addition, we used a simple blood glucose meter, which requires only small blood volumes (10 μL). We confirmed the accuracy of this method by HPLC. The indicated time intervals of incubation after the addition of the test solutions (1, 2 and 3 hours, respectively) were chosen due to the following reasons: First, dependent on the buffer formulation it takes up to 60 minutes until the applied buffer volume is fully absorbed. Thus, earlier time points are not straightforward. Second, the handling of the eggs takes some time. Also a trained researcher is not able to manage more than 10 eggs in the indicated time frame. Therefore, we chose 1 hour time intervals. Third, we are searching for compounds that have a quick effect on blood glucose levels. For this reason we limit our incubation times to 3 hours. ii) We found a prominent effect of the buffer alone (mock control) on the blood glucose concentration, especially in embryos aged 11 days. Therefore, we tested different buffer formulations and conditions. HBSS buffer turned out to be the best suited, while KRPH, PBS and especially HEPES buffer led to an increased reduction of blood glucose levels, thus excluding their application. The definite reason for the varying blood glucose reducing effects of the different buffer formulations remains unclear. We compared the buffer compositions and found significant differences in the osmolarity. Interestingly, HEPES buffer, which has the lowest value of 246,8 mosmol/L, was linked to the largest decrease (35%). All other tested buffers have similar osmolarities between 300–320 mosmol/L, and led to a much lower decrease ranging from 17–25%. In addition, we observed a decrease of blood glucose levels upon buffer application over the analyzed time period (Fig 1B): In comparison to untreated eggs (‘control’), HBSS buffer led to a remarkable decrease after 60, 120 and 180 minutes. We speculate that this could be due to the following reasons: First, a dilution effect (300 μl of buffer was injected in the air bladder and fully absorbed). This seems reasonable since smaller buffer volumes (100 μl) resulted in a much lower decrease.

Second, application of the buffer in the air bladder might be stress-inducing and thereby result in increased glucose uptake and consumption, finally leading to lowered blood glucose levels.

However, experiments based on the application of NovoRapid clearly proved that the effect of blood glucose lowering compounds can be determined in spite of the described buffer effect. Importantly, this effect can be avoided when the absolute amount of the incubated buffer volume is reduced to 100 μL. From the five tested buffers, only HBSS contained a low concentration of glucose (1 mg/L). We are convinced that it does not negatively influence assay performance, as the quantitated embryo blood glucose concentration was found to be more than 1,000 mg/L.

iii) The temperature of the buffer used for incubation of the insulin mimetic compounds does not significantly influence the measurements. Thus, room temperature (23°C in the air-conditioned lab) seems appropriate.

iv) We successfully used the Gluc-HET assay to test the efficacies of putative insulin-mimetic compounds. In our primary screening systems, which is based on the quantitation of GLUT4 translocation [12], we identified several herbal extracts that stimulate GLUT4 translocation in CHO-K1 cells overexpressing the human insulin receptor [29]. Our results clearly show that extracts prepared from *Hedera helix* (ivy), *Papaver somniferum* (poppy) and *Combretum indicum* (Rangoon creeper) did not significantly reduce blood glucose levels. This is in agreement with the current literature, and to our knowledge, there are no known antidiabetic properties of these medicinal plants. However, two additional extracts (0845 and 0846) resulted in a significant and promising decrease. These extracts are currently being investigated in more detail and are good candidates for application in dietary supplements.

v) Finally, the chosen compound application strategy, including the incubation time and concentration of the extracts, did not negatively influence the vitality of the embryos. We quantitated the blood glucose levels of 10 embryos at each time point and for each concentration in at least 3 different experiments. Usually, at most, one of these embryos died during the extract/buffer/NovoRapid incubation procedure. This rate was not higher than the one of the control group. In addition, the condition of the treated embryos was as good as that of the control group. For example, we did not find more lesions or disordered blood vessels, also not upon incubation of the herbal extracts for 24 hours. Thus, the described effects on blood glucose levels are mediated by the tested compounds and not based on cytotoxic effects.

An important point is our chosen strategy for application of the test solutions on the surface of the eggshell membrane. It is different from the HET-CAM system in its original design, which is characterized by the application of compounds on CAM after the removal of the eggshell membrane. Experiences with this application form exist from the HET-micronucleus induction (HET-MN) that has been described in several papers before. We favor this methodology, as it is easier to handle, not prone to a high mortality of the embryos, and, most importantly, ensures a higher response to the test substances as well as better bioavailability than the albumen route [16, 30, 31].

## Conclusion

Taken together, the Gluc-HET system represents a robust method to validate the efficacy of putative insulin mimetic and anti-diabetic compounds in a living organism. It represents a valuable alternative to assays based on live animals. We are convinced that the experimental conditions presented here will contribute to a better acceptance of the assay in the community.
